# Supplementary figures and images for: The induction of preterm labor in rhesus macaques is determined by the strength of immune response to intrauterine infection
Source: PLoS Biol. 2021 Sep 8;19(9):e3001385. doi: 10.1371/journal.pbio.3001385 (PMC8452070; doi:10.1371/journal.pbio.3001385)

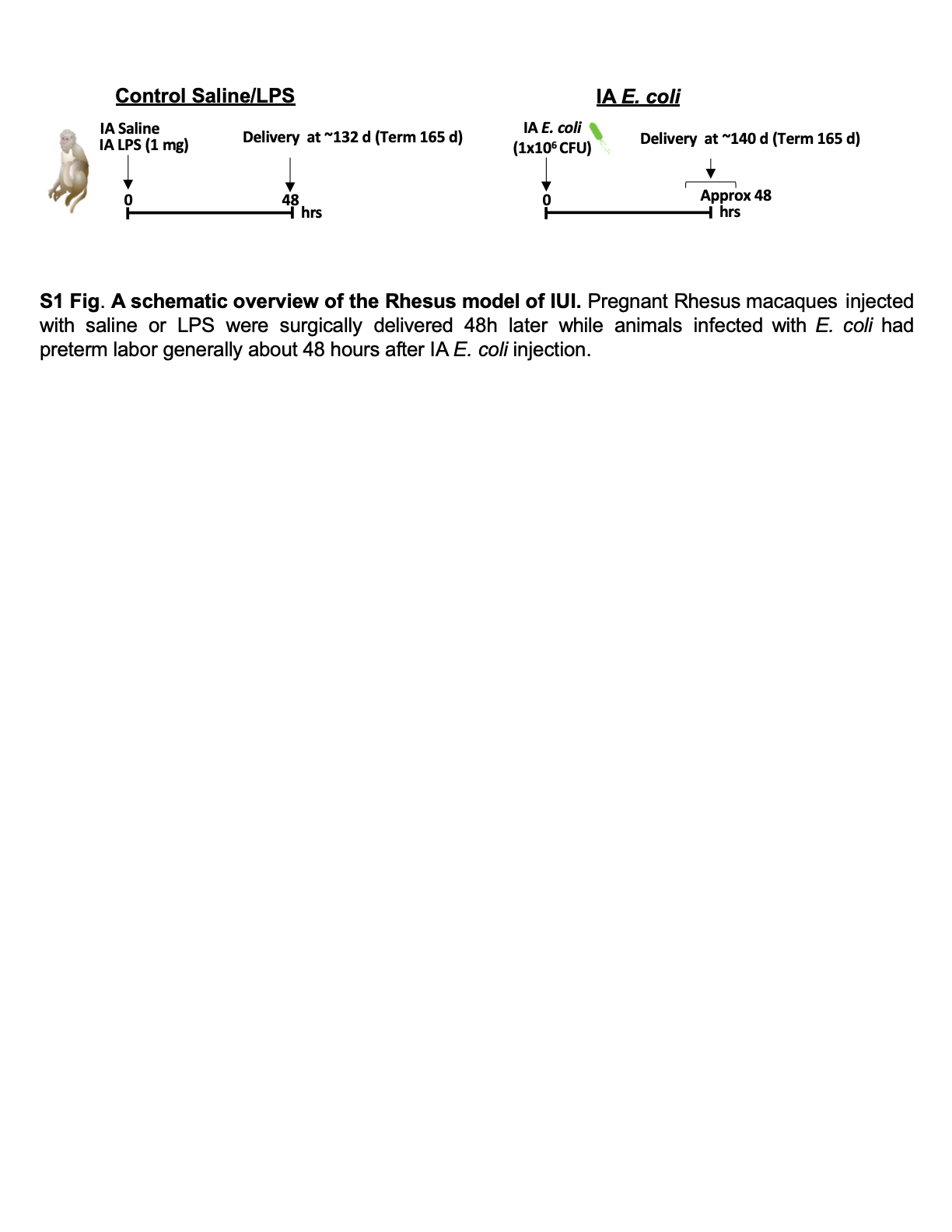

Supplement: S1 Fig — Pregnant rhesus macaques injected with saline or LPS were surgically delivered 48 hours later, while animals infected with E. coli had PTL generally about 48 hours after IA E. coli injection. IA, intra-amniotic; IUI, intrauterine infection/inflammation; LPS, lipopolysaccharide; PTL, preterm labor. (TIFF) [file pbio.3001385.s001.tiff]

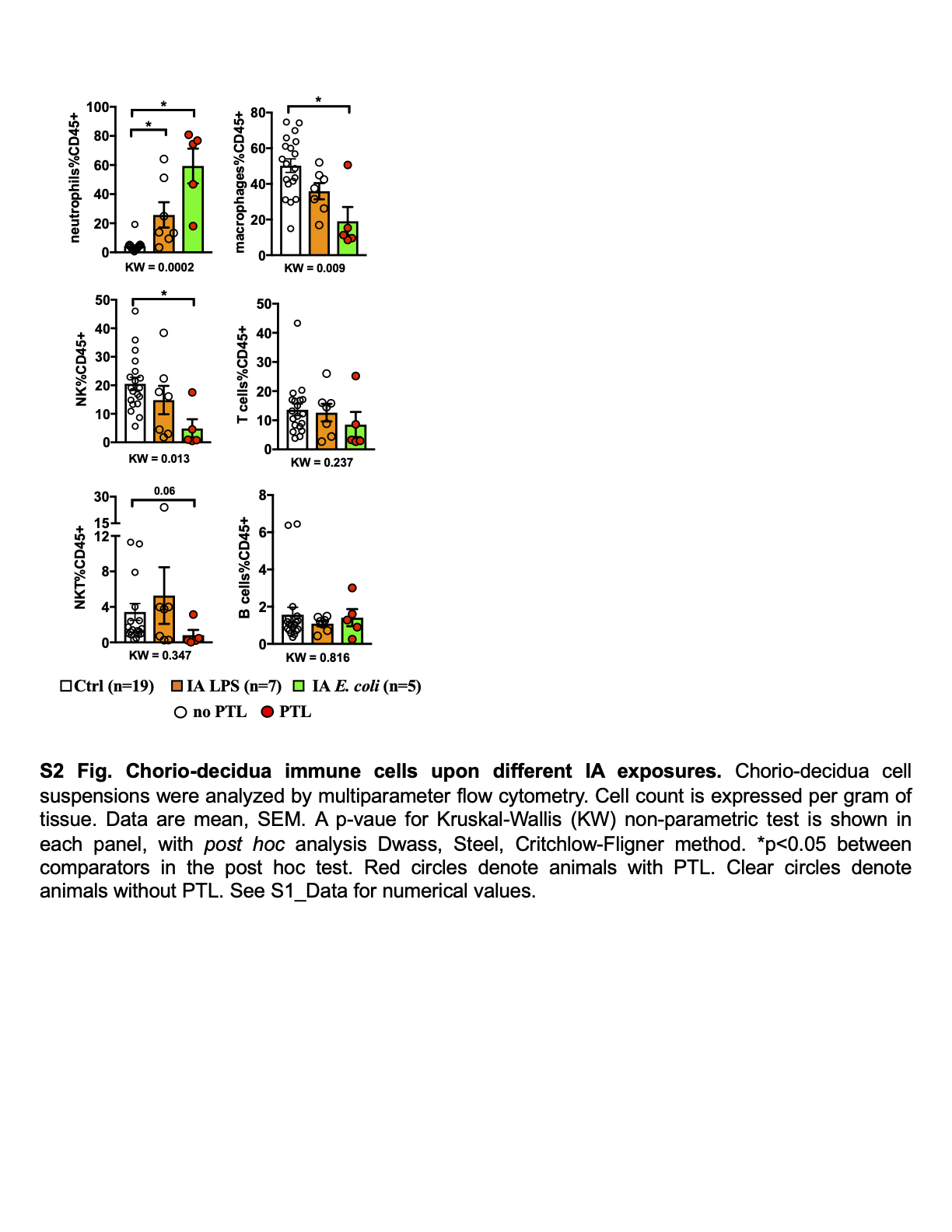

Supplement: S2 Fig — Chorio-decidua cell suspensions were analyzed by multiparameter flow cytometry. Cell count is expressed per gram of tissue. Data are mean, SEM. A p-value for KW nonparametric test is shown in each panel, with post hoc analysis DSCF method. *p < 0.05 between comparators in the post hoc test. Red circles denote animals with PTL, while clear circles denote animals without PTL. See S1 Data for numerical values. DSCF, Dwass–Steel–Critchlow–Fligner; IA, intra-amniotic; KW, Kruskal–Wallis; PTL, preterm labor. (TIFF) [file pbio.3001385.s002.tiff]

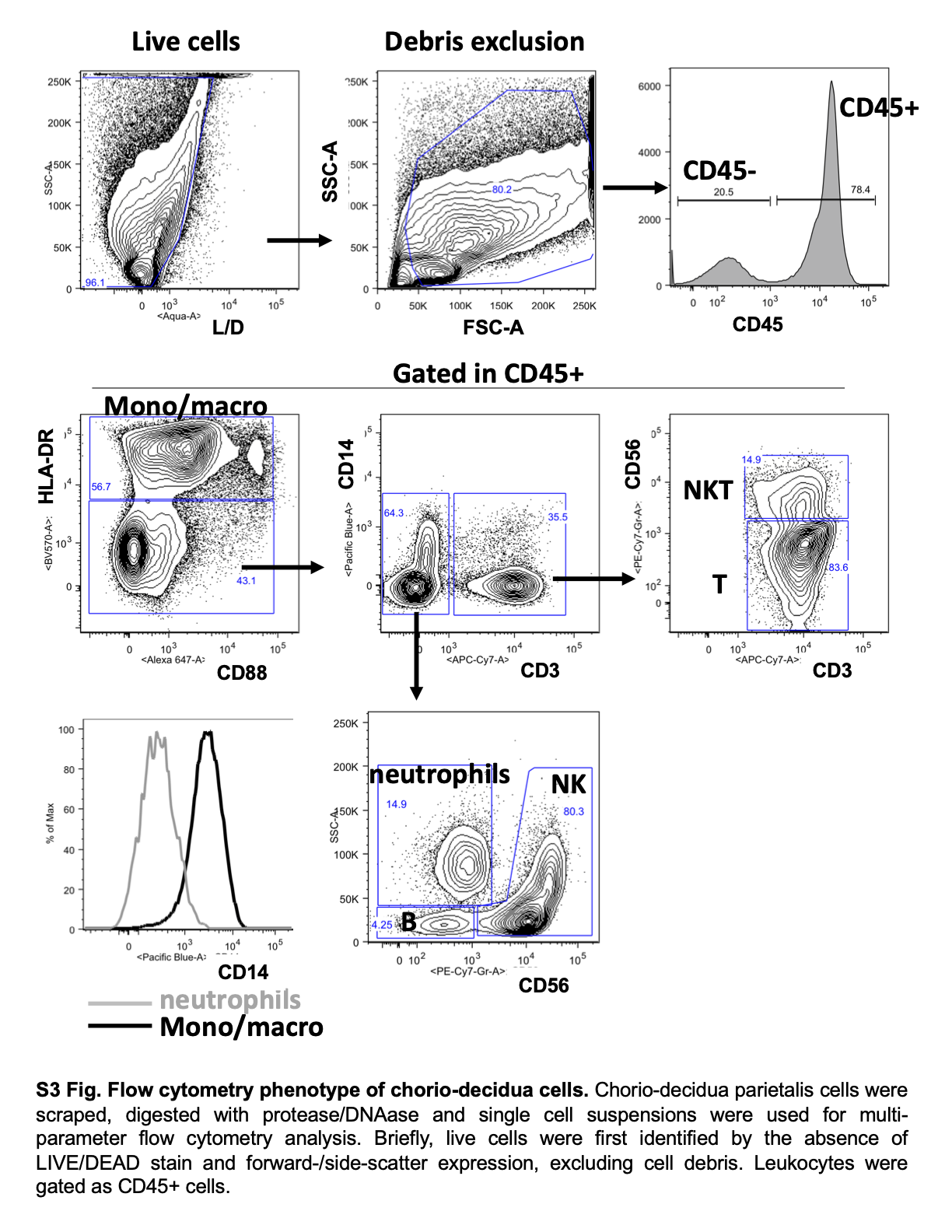

Supplement: S3 Fig — Chorio-decidua parietalis cells were scraped, digested with protease/DNAase, and single cell suspensions were used for multi-parameter flow cytometry analysis. Briefly, live cells were first identified by the absence of LIVE/DEAD stain and forward-/side-scatter expression, excluding cell debris. Leukocytes were gated as CD45+ cells. (TIFF) [file pbio.3001385.s003.tiff]

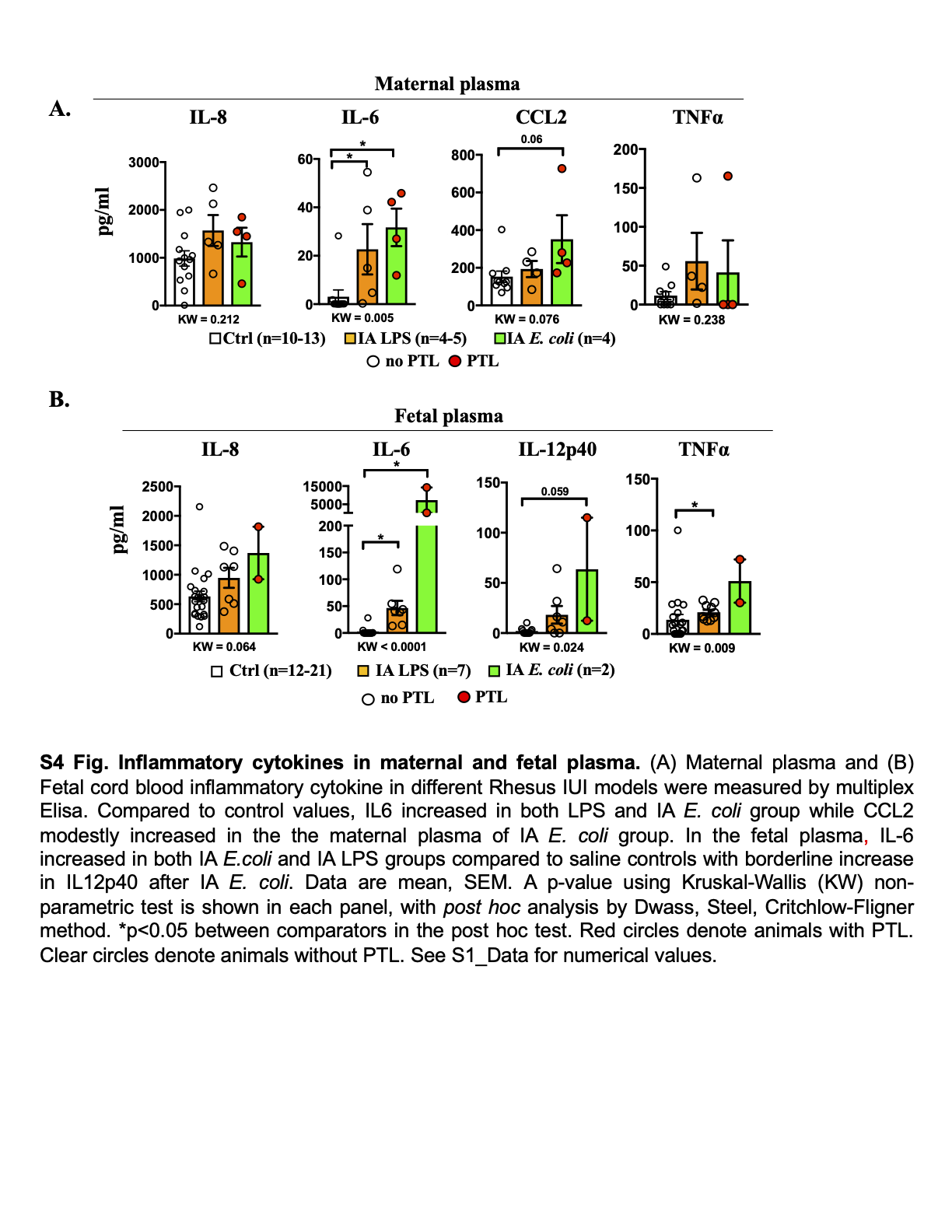

Supplement: S4 Fig — (A) Maternal plasma and (B) fetal cord blood inflammatory cytokine in different rhesus IUI models were measured by multiplex ELISA. Compared to control values, IL6 increased in both LPS and IA E. coli group, while CCL2 modestly increased in the maternal plasma of IA E. coli group. In the fetal plasma, IL-6 increased in both IA E. coli and IA LPS groups compared to saline controls with borderline increase in IL12p40 after IA E. coli. Data are mean, SEM. A p-value using KW nonparametric test is shown in each panel, with post hoc analysis by DSCF method. *p < 0.05 between comparators in the post hoc test. Red circles denote animals with PTL, while clear circles denote animals without PTL. See S1 Data for numerical values. DSCF, Dwass–Steel–Critchlow–Fligner; IA, intra-amniotic; IL-6, interleukin 6; IUI, intrauterine infection/inflammation; KW, Kruskal–Wallis; LPS, lipopolysaccharide; PTL, preterm labor. (TIFF) [file pbio.3001385.s004.tiff]

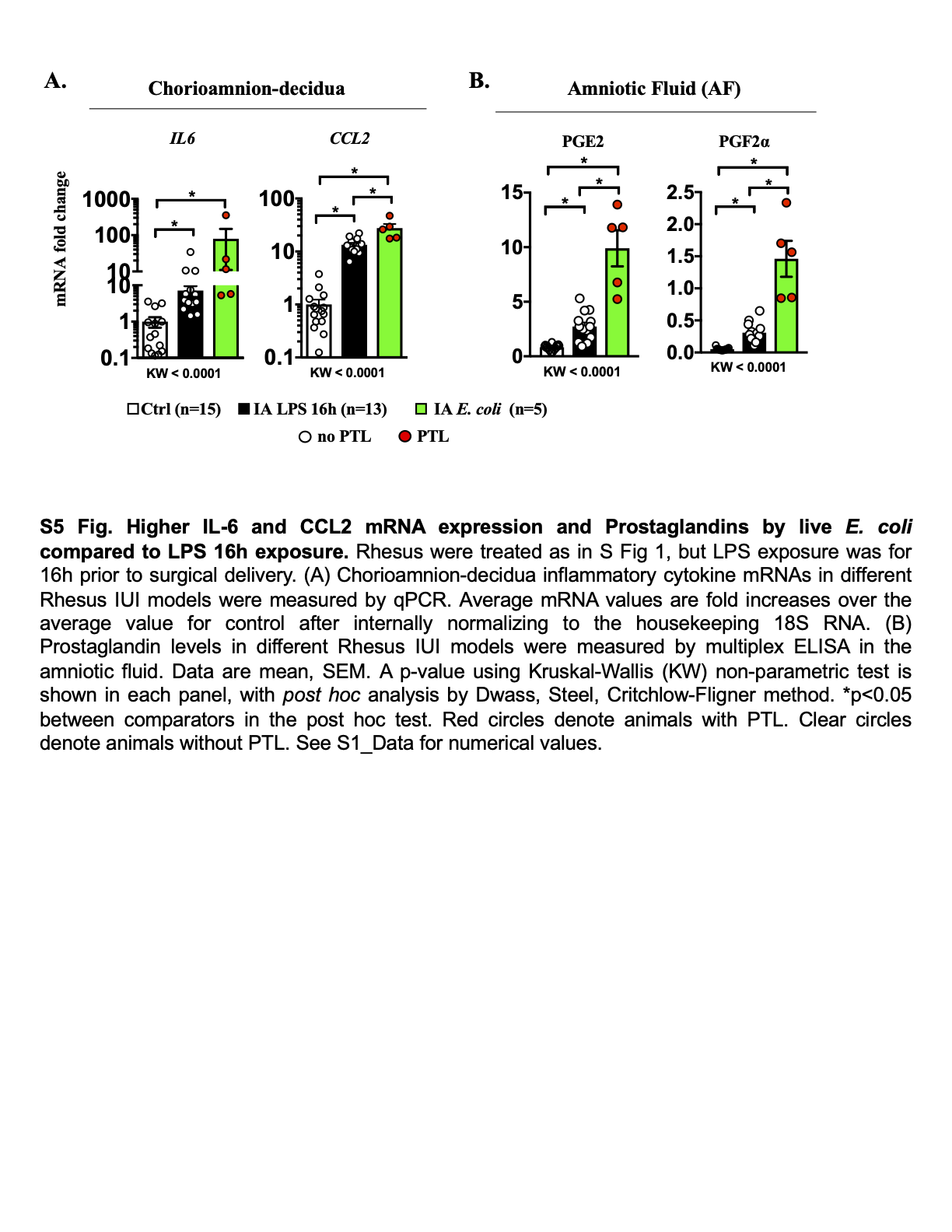

Supplement: S5 Fig — Rhesus were treated as in S1 Fig, but LPS exposure was for 16 hours prior to surgical delivery. (A) Chorioamnion-decidua inflammatory cytokine mRNAs in different rhesus IUI models were measured by qPCR. Average mRNA values are fold increases over the average value for control after internally normalizing to the housekeeping 18S RNA. (B) Prostaglandin levels in different rhesus IUI models were measured by multiplex ELISA in the AF. Data are mean, SEM. A p-value using KW nonparametric test is shown in each panel, with post hoc analysis by DSCF method. *p < 0.05 between comparators in the post hoc test. Red circles denote animals with PTL, while clear circles denote animals without PTL. See S1 Data for numerical values. DSCF, Dwass–Steel–Critchlow–Fligner; IL-6, interleukin 6; IUI, intrauterine infection/inflammation; KW, Kruskal–Wallis; LPS, lipopolysaccharide; PTL, preterm labor; qPCR, quantitative polymerase chain reaction. (TIFF) [file pbio.3001385.s005.tiff]

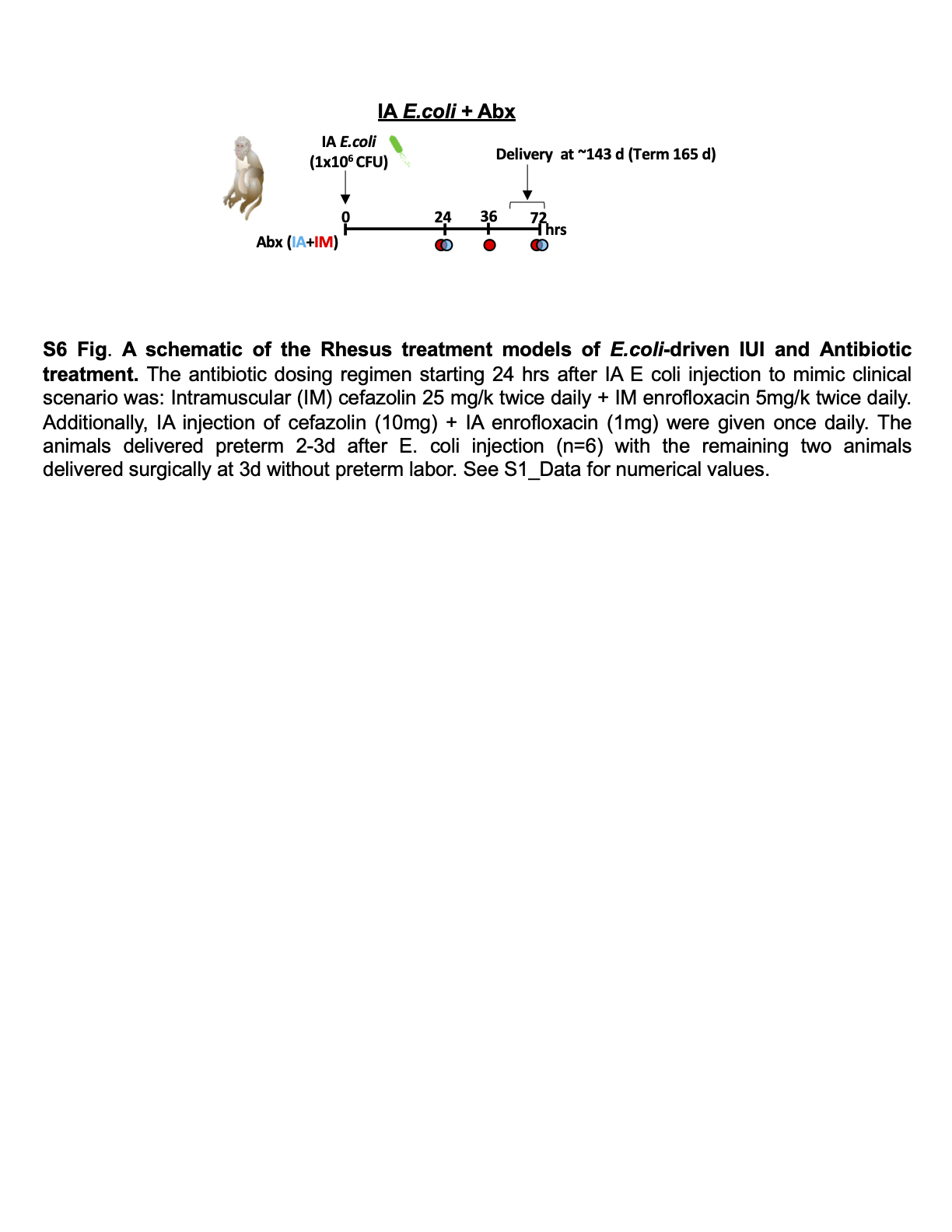

Supplement: S6 Fig — The Abx dosing regimen starting 24 hours after IA E. coli injection to mimic clinical scenario was IM cefazolin 25 mg/k twice daily + IM enrofloxacin 5 mg/k twice daily. Additionally, IA injection of cefazolin (10mg) + IA enrofloxacin (1mg) were given once daily. The animals delivered preterm 2 to 3 days after E. coli injection (n = 6) with the remaining 2 animals delivered surgically at 3 days without PTL. See S1 Data for numerical values. Abx, antibiotics; IA, intra-amniotic; IM, intramuscular; IUI, intrauterine infection/inflammation; PTL, preterm labor. (TIFF) [file pbio.3001385.s006.tiff]

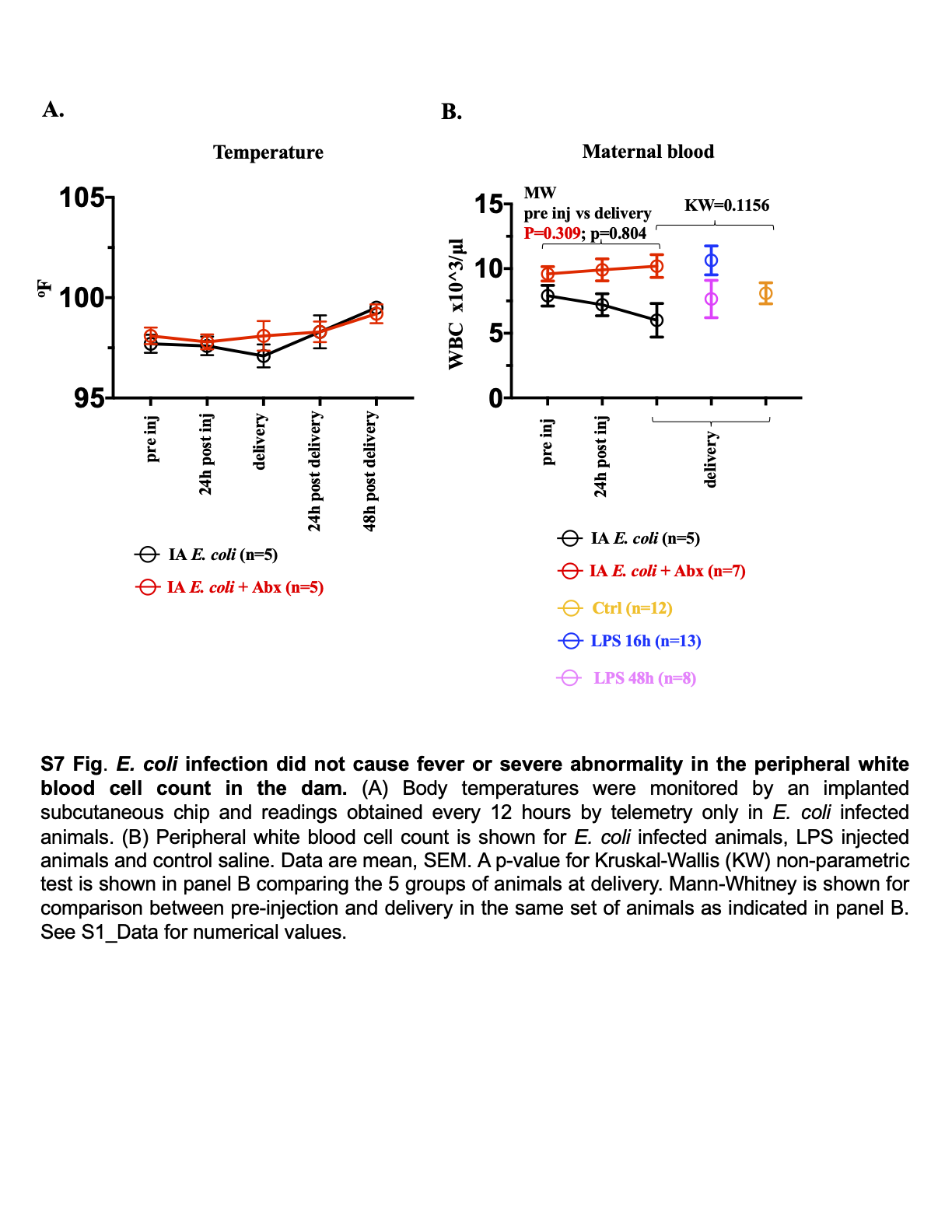

Supplement: S7 Fig — (A) Body temperatures were monitored by an implanted subcutaneous chip and readings obtained every 12 hours by telemetry only in E. coli–infected animals. (B) Peripheral white blood cell count is shown for E. coli–infected animals, LPS-injected animals, and control saline. Data are mean, SEM. A p-value for KW nonparametric test is shown in panel B comparing the 5 groups of animals at delivery. Mann–Whitney is shown for comparison between preinjection and delivery in the same set of animals as indicated in panel B. See S1 Data for numerical values. KW, Kruskal–Wallis; LPS, lipopolysaccharide. (TIFF) [file pbio.3001385.s007.tiff]

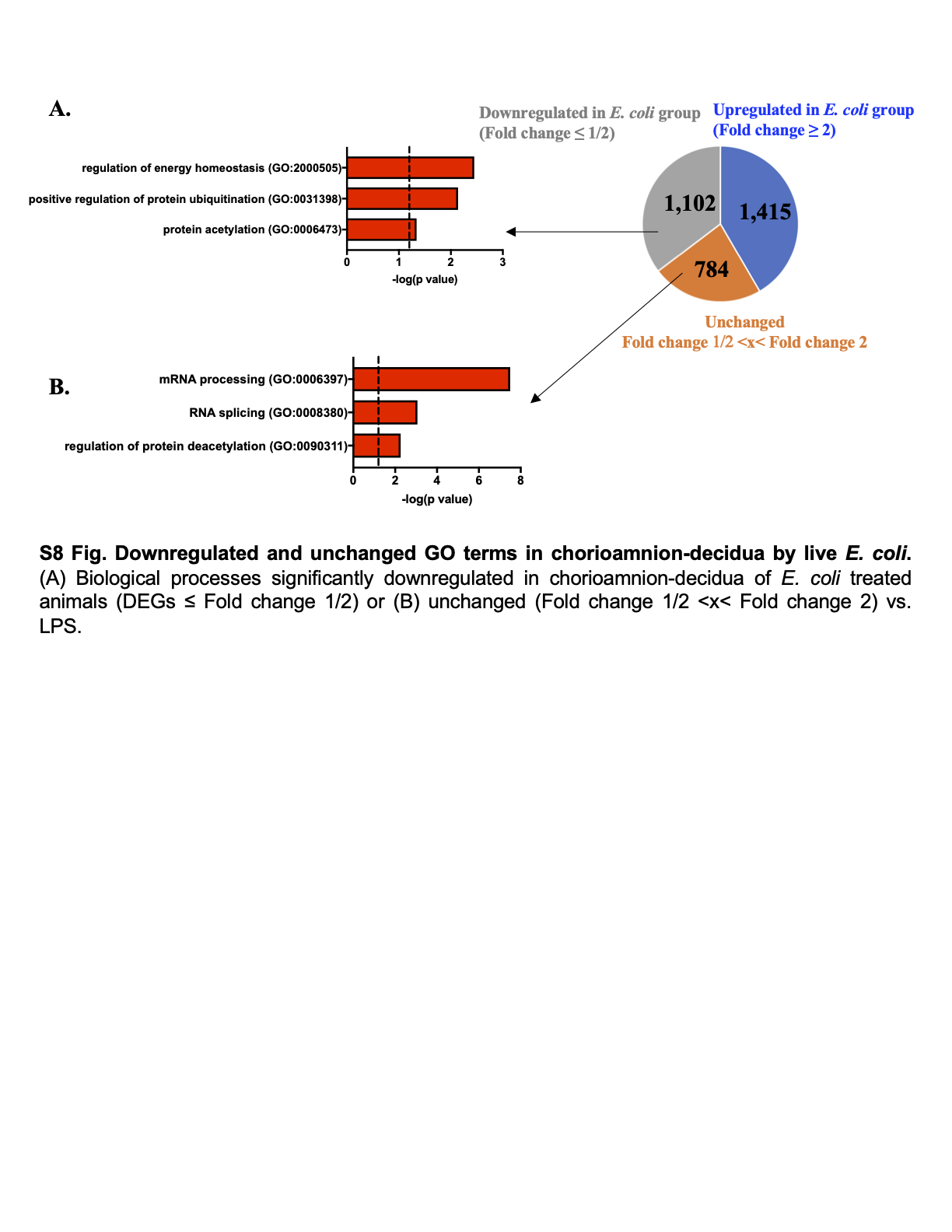

Supplement: S8 Fig — (A) Biological processes significantly down-regulated in chorioamnion-decidua of E. coli treated animals (DEGs ≤ fold change 1/2) or (B) unchanged (fold change 1/2 < × < fold change 2) versus LPS. DEG, differentially expressed gene; GO, gene ontology; LPS, lipopolysaccharide. (TIFF) [file pbio.3001385.s008.tiff]

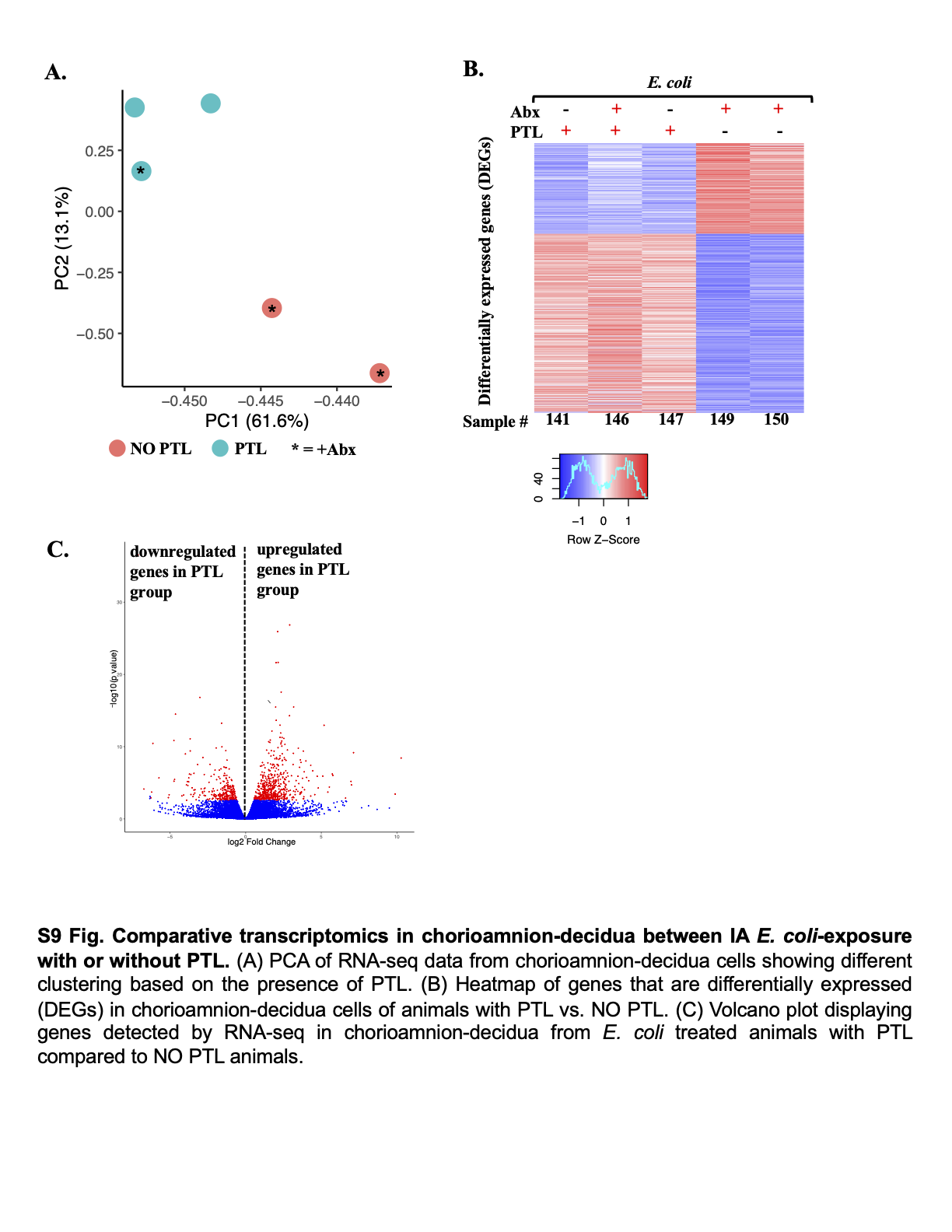

Supplement: S9 Fig — (A) PCA of RNA-seq data from chorioamnion-decidua cells showing different clustering based on the presence of PTL. (B) Heatmap of genes that are differentially expressed (DEGs) in chorioamnion-decidua cells of animals with PTL versus no PTL. (C) Volcano plot displaying genes detected by RNA-seq in chorioamnion-decidua from E. coli treated animals with PTL compared to no PTL animals. DEG, differentially expressed gene; IA, intra-amniotic; PCA, principal component analysis; PTL, preterm labor; RNA-seq, RNA sequencing. (TIFF) [file pbio.3001385.s009.tiff]

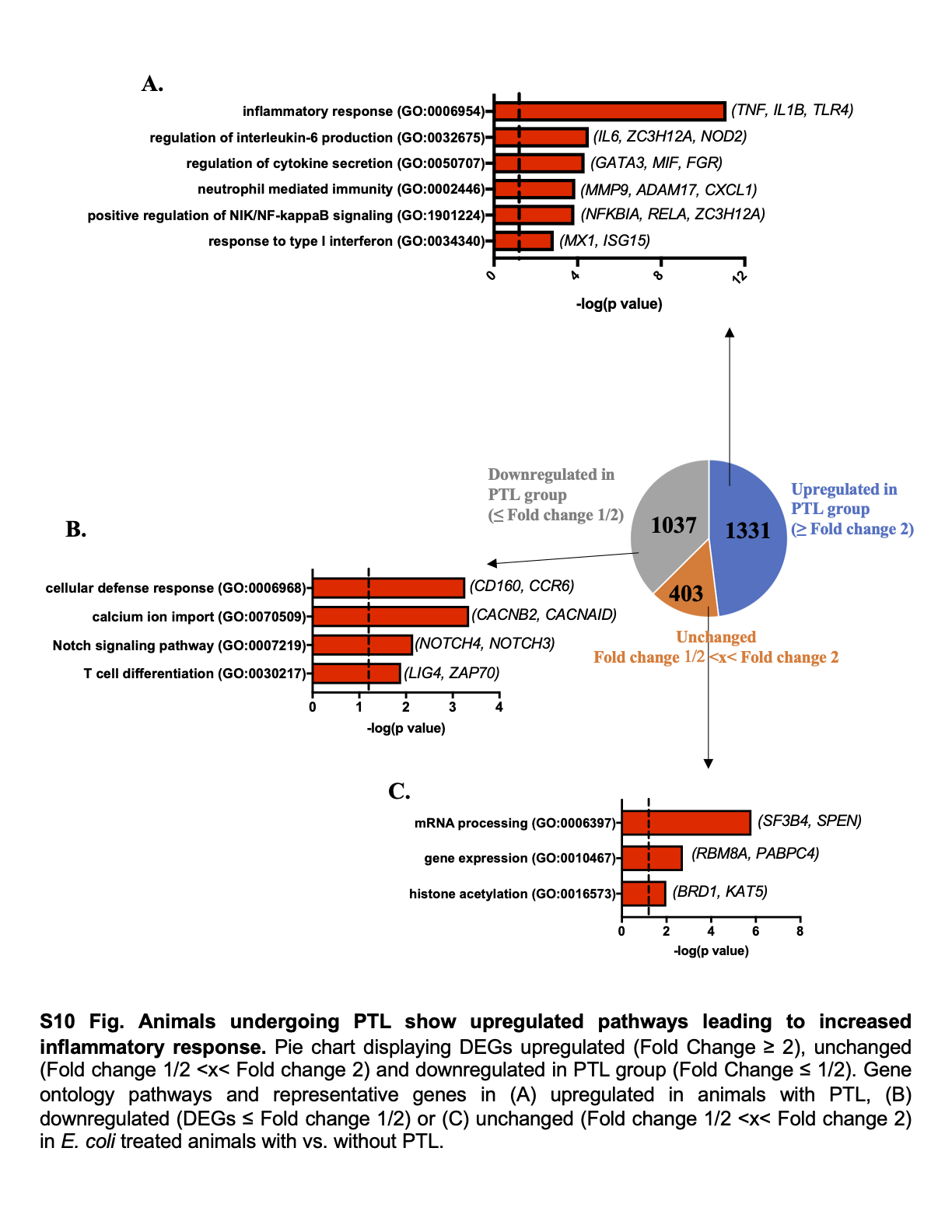

Supplement: S10 Fig — Pie chart displaying DEGs up-regulated (fold change ≥2), unchanged (fold change 1/2 < × < fold change 2) and down-regulated in PTL group (fold change ≤1/2). GO pathways and representative genes in (A) up-regulated in animals with PTL, (B) down-regulated (DEGs ≤ fold change 1/2), or (C) unchanged (fold change 1/2 < × < fold change 2) in E. coli treated animals with versus without PTL. DEG, differentially expressed gene; GO, gene ontology; PTL, preterm labor. (TIFF) [file pbio.3001385.s010.tiff]
