# Supplementary material for: The induction of preterm labor in rhesus macaques is determined by the strength of immune response to intrauterine infection
Source: PLoS Biol. 2021 Sep 8;19(9):e3001385. doi: 10.1371/journal.pbio.3001385 (PMC8452070; doi:10.1371/journal.pbio.3001385)
Supplement: S1 Table — (PDF) [file pbio.3001385.s011.pdf]

S1 Table. Animal data and sample sizes.

	**Ctrl**	**LPS 16h**	**LPS 48h**	***E. coli***	***E coli* + Abx[1]**
n (total)	21	13	8	5	8
n for sequencing	3	-	3	3	2
n for protein levels	6-21	-	4-7	4-5	5-6
n for RT-PCR	7-15	13	6-8	4-5	4-6
n for flow cytometry	14-19	-	7	5	6
n for RNAscope+IF	3	-	4	5	3
Gestational age at delivery (days, mean±SD)	132±2	131.9±3	132±4	140.3±5	143±4
Maternal weight (kg±SD)	8.9±1.5	8.1±1.7	8.4±1.1	8.8±2.1	10.0±1.2
Fetal weight (g±SD)	331±7	320±38	320±45	356±45	397±57
Sex (M/F)	13/8	5/8	1/7	3/2	3/5

[1] Abx=Antibiotics

Note: it was not always possible to obtain all the tissues/fluids from each animal. The numbers of animals for each experiment are also shown in the corresponding figure.
