# Supplementary material for: The induction of preterm labor in rhesus macaques is determined by the strength of immune response to intrauterine infection
Source: PLoS Biol. 2021 Sep 8;19(9):e3001385. doi: 10.1371/journal.pbio.3001385 (PMC8452070; doi:10.1371/journal.pbio.3001385)
Supplement: Attachment — Submitted filename: Response to reviewers_FINAL.docx [file pbio.3001385.s013.docx]

Editor’s comments:

1) Having discussed the reviews with the academic editor, we think that it is important that you clearly describe your experiments and clarify the number of animals that were used.

**We would like to begin by thanking the reviewers and the editor for providing detailed and constructive remarks response to which, along with our new data, have greatly improved our manuscript. Notably, the edited version of our manuscript, in addition to significant changes in the text, consist of eight Main Figures ten Supplementary Figures, two Tables and one Supplementary Table.**

**Detailed responses to specific comments raised by the reviewers are provided below.**

**We have now added 2 schematic timelines to improve the understanding of the experimental design and the number of animals used in the study (Supplementary Figs 1 and 6)**

2) Also, regarding the statistics performed, you could do a statistical transformation of your data and then use a parametric method. Given the unequal numbers in the groups it might be better to use GLM rather than conventional ANOVA, as reviewer #2 suggests, but we strongly suggest you to ask for proper statistics advice.

**We have added Dr. Myung Sim, an experienced Bio-statistician as a co-author. All the statistics have been re-analyzed. Considering the small sample sizes, the normality of the distributions was (or would be) hard to reach even after the transformation. The main analysis was univariable analysis in which case ANOVA and GLM would give the exact same results. Considering these facts, we tested overall difference in 3 groups using nonparametric Kruskal Wallis test and used Dwass, Steel, Critchlow-Fligner (DSCF) multiple comparison adjustments for post hoc pairwise comparisons.**

3) Reviewer #1 thinks that the data presentation is confusing and suggests to add a table or flow chart that presents a clear experimental scheme.

**As suggested, we have added two schematic timelines (Supplementary Figs 1 and 6)**

4) This reviewer also says that figure 6 as it stands does not support the hypothesis, asks why was fetal plasma collected from only two fetuses,

**Please refer to response to Reviewer #1 comment #6 below. As regards fetal plasma, some dams had preterm vaginal delivery or intrauterine fetal demise. When these cases were discovered, we did our best to collect post mortem tissues when possible. Unfortunately, it was not possible to collect blood from these cases as mentioned in Supplementary Table 1.**

5) thinks that you should provide additional assurance that the only difference between the LPS and the E. coli treatment is intensity of the inflammation.

**Please see response to reviewer #1, Comment #8**.

6) Reviewer #2 thinks that you should discuss how the initial dose of E. coli or bacterial proliferation post-inoculation equates to the dose of LPS used in the study and review other data on the effectiveness of bacterial infection and LPS inducing preterm labour in animal models including non-human primates in the intro.

**Please see response to reviewer #2, Comment #3.**

7) This reviewer also has questions about the maternal weight in the different groups.

**Please see response to reviewer #2, Comment #8.**

8) says that the estimation of immune cell infiltration by H&E staining is insufficient,

**In light of the reviewer’s comments, we have now added images showing MPO staining in the chorioamnion-decidua (Figure 1A).**

9) the representation of the data in fig 1B is misleading, and is not convinced of the robustness of the data. Reviewer #2 has also further comments that you will need to address.

**As suggested by reviewer #2, we have revised Figure 1B to show the absolute counts rather than % cells which could be misleading as suggested by the reviewer.**

Reviewer #1:

1) This is a comprehensive study, which, for the most part, tells a logical story.

**We thank the reviewer for the constructive comments. Our point-by-point responses are provided below. The revised text is indicated by red font in the manuscript.**

2) However, the way the data are presented is confusing. Based on Table 1 there are 5 animals that were administered E. coli. and they all developed PTL. However, in other parts of the paper, it appears that there were 8 animals injected with E. coli.

**We thank the reviewer for this important comment and apologize for the confusion.**

**We have now corrected table 1 and supplementary table 1 indicating precise animal number and group interventions. We first used 5 animals infected with *E.coli* (5/5 went into preterm labor). To control bacteremia and thus simulate clinical situations, we added another group of 8 animals infected with *E.coli* and treated with antibiotics (6/8 went into preterm labor). We have revised the results indicating that animals infected by *E. coli* and treated with antibiotics (Abx) are a separate group of 8 animals (lines 231-238 and Supplementary Table 1).**

3) For example, I don't understand these two sentences in the Methods:

The animals given antibiotics delivered preterm 2-3d after E. coli injection (n=6) with the remaining two animals delivered surgically at 3d without preterm labor

**As mentioned in response #2 above, these animals belong to the second set (*E.coli* infection followed by Abx treatment; 6/8 went into preterm labor).**

4) Only 1 animal in the E. coli group delivered vaginally and the remainder [sic] 4 animals were surgically delivered in active preterm labor. So, were there 5 or 8 animals treated with E. coli? Did they all develop PTL or not? Table 1 is different from Supplementary Table 1.

**As mentioned in response #2 above, these animals belong to the first set (*E.coli* infection only). All 5 went into preterm labor. 1 had vaginal delivery and 4 were delivered by C section after active labor was confirmed.**

5) I think one table or flow chart that presents a clear experimental scheme would greatly strengthen this manuscript.

**We agree. We therefore added a flow chart for each experimental model Supplementary Figure 1 and 6).**

6) Is there a mistake in Figure 6? Didn't all 5 E. coli challenged animals develop PTL? Figure 6, as it stands, does not support the hypothesis. But Figure 6 is different from Supplementary Figure 6.

**Figure 6 transcriptomic analysis includes 2 animals infected with *E. coli* only (2/2 had PTL) and 3 animals infected with *E.coli* and treated with Abx (1/3 had PTL, 2/3 had NO PTL). As shown in Figure 4, Abx treatment did not impact the extent of *E. coli* induced inflammation. The main goal in Figure 6 is to determine the *E.coli*-specific pathways compared to LPS-driven inflammation. In Supplementary Figure 9, we compared samples that had or not PTL after *E. coli* infection with or without Abx treatment. The main goal in Supplementary Figure 9 is to determine the labor-specific pathways upon *E. coli* infection. Since we had only 2 animals without PTL in this group, we chose to show the data as a Supplementary rather than a Main Figure.**

7) In Supplemental Figure 3B, why was fetal plasma collected from only two fetuses?

**Unfortunately, there were some missed samples and it was not always possible to obtain all the tissues/fluids from each animal, in particular when vaginal delivery or intrauterine fetal demise occurred.**

8) Additional assurance that the only difference between the LPS and the E. coli treatment is intensity of the inflammation is needed. In other words, since most of the E. coli challenged macaques developed bacteremia, although it was treated, can we be sure that these bacteremic animals did not have signs and symptoms of sepsis? In other words, might the bacteremic animals have had systemic changes, such as hypoxia, decreased perfusion, metabolic acidosis, etc., which could explain increased rates of PTL? Please provide data showing the absence of clinical stigmata of sepsis or changes in clinical status in the E. coli vs. LPS treated macaques.

**The reviewer raises an important question. We have added a new Supplementary Figure 7 showing body temperatures and blood counts in Rhesus macaques infected with *E. coli*. Body temperatures were monitored by an implanted subcutaneous chip and readings obtained every 12 hours by telemetry without the need for handling or sedating the animal. As can be seen from the data, these animals did not develop significant fever or severe abnormality in the peripheral white blood cell count. These additions are now shown in the revised material and methods (lines 485-490) and results (Supplementary Figure 7). In addition, these animals were closely monitored by trained veterinary personnel at the California National Primate Center. They did not report clinical signs of illness including lack of appetite, changes in feeding, activity level, vomiting or diarrhea. There was no maternal mortality in this cohort. Thus, we feel that clinical stigmata of sepsis did not significantly confound the analyses.**

Reviewer #2:

1) Overall: This is a well-written, interesting study which addresses mechanisms underlying inflammatory-mediated preterm labor. The study addresses discrepancies by which bacterial infection but not LPS can induce preterm birth in various animal models. The authors suggest that this can be explained by the degree of inflammatory response generated by these agents, and that the greater inflammatory response generated by bacterial is required to induce preterm labor.

**We thank the reviewer for his/her constructive comments. Our point-by-point**

**responses are provided below. The revised text is indicated by red font in the manuscript.**

2) The manuscript requires revision to address the points raised below. Two major issues should be addressed. Firstly, the use of Mann-Whitney U-test for 3 groups is not appropriate and the applicants should consider other tests such as ANOVA. This is important as it may reduce the statistical significance of the data measures.

**We agree. We tested overall difference in 3 groups using nonparametric Kruskal Wallis test and used Dwass, Steel, Critchlow-Fligner (DSCF) multiple comparison adjustments for post hoc pairwise comparisons. Overall, the interpretation did not change significantly although a few data points that were previously significant, are now not significant (as in Fig 5).**

3) Secondly, the study seeks to compare the effectiveness of bacterial inoculation with LPS administration. However, the authors do not discuss the how the initial dose of E coli (1*106 CFU) or bacterial proliferation post inoculation equates to the dose LPS (1 mg) used in the study.

**The reviewer raises an important question. It is difficult to compare the doses of a live organism that is rapidly multiplying to a non-replicating ligand. We previously published in a sheep model that the half-life of LPS given by intraamniotic injection is about 29h (Newnham, J.P et al *American journal of obstetrics and gynecology* 189, 1458-1466 (2003). PMID 14634586). In contrast with LPS administration, injection of live *E. coli* increases host exposure microbial products dynamically with time. As a rough proxy, we have shown comparative Endotoxin levels in the amniotic fluid to be about 3-fold higher in the live E. coli group at the end of the experiment (Figure 2B). Since the exposure levels vary dynamically during the experiment over time in both the models, we feel a better gauge of the relative exposure levels is the host immune response. Since many of the read outs of inflammation are comparable, we feel the overall comparison of both models is justified. We have added a consideration of the dose responses in the revised discussion and also mentioned this as a possible limitation of the study (Lines 451-463).**

4) Murine studies show that different doses of LPS and different LPS preparations injected into pregnant dams at late gestation cause utterly different effects.

**We agree that murine studies do show significantly different effects with different doses and preparations of LPS. The dosage used in our study were based on non-human primate (NHP) studies in other labs and our prior extensive work in sheep models. Sheep maternal weight, amniotic fluid volume, and fetal weights are approximately 7-10 fold higher than Rhesus macaques. We did extensive dose titration studies (0.1 to 20 mg) in the sheep with intraamniotic injection of O55:B5 *E.coli* derived LPS (same preparation used in our Rhesus studies). We reported that the minimal dose for consistent amniotic inflammatory response was 4mg in the sheep. (Kramer et al *American journal of respiratory and critical care medicine* 164, 982-988 (2001). Kallapur et al *American journal of physiology. Lung cellular and molecular physiology* 280, L527-536 (2001)). There was a plateau response after 10 mg dose in the sheep. Previous NHP studies showed amniotic inflammatory response to 0.01 to 0.1 mg LPS (Adams Waldorf, et al *Reproductive sciences* 15, 121-127 (2008)). The LPS preparation used by Adams Waldorf et al was *E. coli* O111:B4 in a chronically instrumented and surgical Rhesus model. Extensive dose finding experiments is not feasible in NHP. Based on allometric dose scaling from sheep and previous studies in NHP (albeit instrumented model), we aimed for a dose at the higher end to simulate plateau phase of a theoretical dose response curve. We have validated our model for both consistency from year to year for the past 7 years and also with human data. We previously published that the 1mg O55:B5 LPS intraamniotic dose in the Rhesus macaque produces intrauterine inflammatory response that is very similar to human subjects with histologically diagnosed chorioamnionitis (Presicce, et al *JCI Insight* 3 (2018). Based on these considerations, we feel that our model has reproducible responses with inherent biologic variability (lines 75-99).**

5) Introduction

The introduction is quite short and should review other data on the effectiveness of bacterial infection and LPS inducing preterm labour in animal models including non-human primates.

**We thank the reviewer for the comment. We have now added a paragraph incorporating points from #4 above (Lines 75-99).**

6) Results

For instance, how the dose of E coli ( 1*106 CFU) correspond to LPS ( 1 mg)? Mouse studies show that different doses of LPS and different LPS preparations injected into pregnant dams at late gestation cause utterly different effects.

**Please refer to response to #3 above.**

7) Maternal weight: I assume "in grams" please add

**We thank the reviewer and apologize for omitting the unit (kg) in table 1.**

8) Table 1: Is there any significant difference in Maternal weight at 48h and 5 days (LPS group) or with the E coli group? Any comments??

**Unfortunately, at the time these experiments were done, maternal weights were not done prior to surgical delivery at the end of the experimentation. These procedures have now been revised. Thus, we cannot definitely comment on the weight difference. However, these animals were carefully monitored for health by trained veterinary personnel. The appetite and food intake die not change during the exposures to inflammatory agents or infection. Furthermore, peripheral blood white blood counts did not change significantly for the duration of the experiment (Supplementary Figure 7B). Animals infected with E. coli did not develop significant fever (Supplementary Figure 7A). There was no maternal mortality in these experiments**.

9) Line 100, Table 2: AF culture - why only three animals were analyzed? ('3/3")

**Unfortunately, it was not always possible to obtain all the tissues/fluids from each animal, in particular when vaginal delivery occurred.**

10) What happened to CD culture? not detected and not analyzed?

**We have clarified that NA in Table 2 refers to “not analyzed”.**

11) Figure 1A: Estimation of immune cell infiltration by H&E staining is insufficient. Please provide a specific IHC evaluation for neutrophil infiltration as it is done in Figure 5 (i.e. MPO or other available neutrophil markers)

**In light of the reviewer’s comments, we have now added an evaluation of neutrophil by MPO staining (Figure 1A).**

12) Figure 1B: The figure is slightly misleading - the absolute count of macrophages, NK, T-cells and B cells does not change after E coli vs LPS. ONLY the neutrophils are dramatically increased, which is a logical response to acute infection at 48 hours. I would rather present an absolute count (or counts per unit weight as in supplementary material), not % of CD45.

**In light of the reviewer’s comments, we are now showing the absolute count in Figure 1B and the frequency in Supplementary Fig 2.**

13) Line 150, Figure 2B: This "trend " is due to 1 or 2 samples (from 5); the other 3-4 are not different from the control, I'm not convinced on the robustness of these data. Indeed, in the majority of figures the current statistical analysis shows significance only between E. coli and control groups.

**We agree. Further, after nonparametric Kruskal Wallis test and DSCF for post hoc pairwise comparisons, there is no statistical difference between the *E. coli* vs LPS groups for AF neutrophil counts (Figure 2C) and all cytokines (Figure 2D), although the PGE2 and PGF2A levels are different between the two groups. We have therefore revised to replace the word “trend” in line 150 (now line 177) to “not statistically different”.**

14) Line 172-175 - This is not a sentence and should be rewritten for clarity.

**In light of the reviewer’s comments, we have rewritten the sentence (new line 183).**

15) Line 178: P values are shown between TWO groups, while there are THREE groups studied. One-way ANOVA should be used, not T(Mann-Whitney U)-test. Same comment for figure 3

**We agree. Please see response to Editor #2.**

16) Lines 200-209: Text describes 8 subjects treated with Abs, 6 delivered preterm. Why are only 4-5 are shown on Figures A,B,C?

**We have now shown the n for each assay in addition to the total number of subjects in each group (Supplementary Table 1). The question referred to in lines 200-209 of the previous submission relates to Fig 4 (Comparison of *E. coli* with and without antibiotics for immune response readouts). The lower number of samples is for amnion tissue which was not possible to separate cleanly in all animals. In addition, in some animals some fluids/were either not collected properly or not available because of multiple assays exhausted the sample. We wish to clarify that all the assays done have been reported and that no data have been withheld.**

17) Lines 220-221: "… IL6 and PGs were the key differentially expressed genes…" - Could the authors clarify what dataset they are referring to for this statement, as it stands this is not clear.

**Data referred to Figure 2A (IL6 and PTGS2 gene expression in chorioamnion decidua). In light of the reviewer comment, we have now specified the dataset in the manuscript (line 168-169).**

18) Line 222: Very interesting data - please describe this methodology - I could description of the RNAscope® fluorescence in situ hybridization in the manuscript.

**In light of the reviewer’s comments, we have added a brief description of dual RNAscope®/immunohistology technology (Lines 261-265).**

19) Line 262, Figure 6A : - What analyses were conducted to define clustering? While E.coli animals seem closer together has the limits of the clusters been defined.

**Figure 6A and Supplementary Figure 9 show that dimension reduction along 2 principle components (PC1 and PC2) were sufficient to separate the three groups. While control and *E. coli* groups were separated in both PC1 and PC2 axes, LPS vs *E. coli* were mainly separated along PC2 axis. Clustering refers to visual separation of the transcription profiles along PC1 and PC2 in principal component analysis. We have deleted the word “clustering” and instead used the word “segregated” in describing the PCA results (line 307). We show below a graphic representation of the PC values of gene expression for the different groups.**

**Figure 1: box-and-whisker plots (below) showing the distribution of A) Ctrl (red), LPS (blue), and *E coli* +/- Abx (green) and B) of *E. coli* NO PTL (red) and *E. coli* PTL (blue) in PC space.**

20) Line 297 : again should consider use of One-way ANOVA

**We agree. We have used nonparametric Kruskal Wallis test and used Dwass, Steel, Critchlow-Fligner (DSCF) multiple comparison adjustments for post hoc pairwise comparisons. Details are given in response #2 to the Editor.**

21) Discussion

Line 336: How do the author explain a significant difference in the neutrophil infiltration without changes in the IL8 expression, and while there is no change in the monocytes, CCL2 is upregulated?

**While the chorio-decidua IL8 mRNA and amniotic IL8 protein levels were not significantly different between the LPS and live *E. coli* groups, IL8 mRNA expression was significantly higher in the amnion of *E.coli* group compared to LPS (Figure 3). We think that one key difference between LPS and *E. coli* group was neutrophil infiltration, activation in the amnion tissue (Figures 3 and 5). Furthermore, there are other neutrophil chemoattractant beyond IL8 that were not all measured here. Chemoattractant upregulation would be expected to precede neutrophil influx. Because we did not have chronically catheterized model, we could not collect fluids at early time points. As regards monocytes, decidua tissue is rich in macrophages at baseline conditions. As can be seen in revised Figure 1B, chorio-decidua macrophage pools do not significantly change between controls and the two experimental groups. In general, tissue macrophages in many anatomical sites do not have significant turn-over. While blood monocyte recruitment is one strategy, there are other complex factors regulating the macrophage pool. Thus, we appreciate the question regarding CCL2 raised by the reviewer, but feel that mechanistic determination of decidua macrophage biology is beyond the scope of this manuscript.**

22) Lines 370-372 : these data (re: NFkB) are not shown in this manuscript - Please add the data or correct the Discussion.

**We have now edited our statement in the Discussion (Line 415-417) to mention IL6 and type I responsive genes (shown in Figure 2 and Figure 7).**

23) Line 379 missing 1 in "Type 1"

**We have corrected the typo (line 423).**

24) Lines 405-412: I think this paragraph could be strengthened. For example "potentially provide possible explanations" and "study provides further insights" are not very informative

**We thank the reviewer for the comment. The paragraph has been modified to make it more informative (line 470-471).**

25) The study Strengths and Limitations part is missing.

**We thank the reviewer for the comment. We have added the Strengths and Limitations paragraph in the discussion (Lines 451-463).**

26) Methods,

I couldn't find details of the animals treated with LPS other than that they were previously reported in another study. It would help to provide some details here so that the reader can assess interpret the presented data.

**Information regarding LPS treatment is described in line 486, 493-496. Some controls and all the LPS 16h and 48h animals were used in previous studies (Presicce, 2018; 2020), while all the E coli infection animals have not been previously reported. We have also added a timeline in Supplementary Figure 1.**

27) lines 422-426: The authors state that treatment with antibiotics reduced bacteremia but it is not clear how this was confirmed.

**Maternal blood and amniotic fluid cultures were drawn before the start of antibiotics (1d after IA injection of *E. coli*) and 1d after start of antibiotics (or 2d after IA E. coli) and at the end of the experiment. Fetal blood culture was drawn at the end of the experiment. We have clarified this both in results (Line 233-237) and in the methods (Lines 485-490).**

28) Lines 422, 426-429 - it is hard to understand the number of animals used: Suppl Table 1 shows 7 (not 8) animals, E coli group n=6, but in the text - 4+1.

**We apologize for the mistake. We have now edited Supplementary Table 1.**

29) Line 446: Trypan blue is not the best method to determine viability when using cells for FACS analyses.

**For FACS analysis, LIVE/DEAD Fixable Aqua dead cell stain (Life Technologies) was used to exclude dead cells form the analysis (line 541). Trypan blue was used to check viability after enzymatic digestion.**

30) Line 523: The authors discuss conducting H&E staining on human fetal membranes. I can't find mention of this in the results and not sure what relevance of such analysis in the current study.

**We apologize for the mistake. We have now deleted the reference to human H&E.**

31) Line 529-534, Mann-Whitney U test for comparisons with multiple groups is not appropriate, the authors should consider using ANOVA.

**We agree. Please see response to Editor #2 for details.**

32) Line 537: It is usual to provide more details regarding the REB #, etc

**We have added the Animal Welfare IACUC approval number in the methods (line 617).**
