# Supplementary material for: The induction of preterm labor in rhesus macaques is determined by the strength of immune response to intrauterine infection
Source: PLoS Biol. 2021 Sep 8;19(9):e3001385. doi: 10.1371/journal.pbio.3001385 (PMC8452070; doi:10.1371/journal.pbio.3001385)
Supplement: Attachment — Submitted filename: Response to reviewers (PBIOLOGY-D-21-00132R2) .docx [file pbio.3001385.s014.docx]

PLOS Biology Submission (PBIOLOGY-D-21-00132R2) - [EMID:f8a280468aa041ed]

Response to reviewers

Reviewer remarks:

Reviewer #1: Sandra E. Reznik, M.D., Ph.D. Reproductive immunobiology.

Reviewer #2: Mechanisms responsible for the onset of labour.


Reviewer #1: The revised version of the manuscript addresses essentially all my previous concerns.

Regarding the basic protocol and animal groups, the revisions and corrections have resolved most of the inconsistencies previously present. I have one remaining small issue there. I don't see where the third group in Table 1 (LPS/ 5 day exposure) is mentioned in either the text or Supplementary Figure 1. Is this group part of the current study?

We agree that it is confusing, and we thank the reviewer. This group of animals was used only to evaluate whether an extended IA-LPS exposure (i.e. 5 days) results in PTB. In other words, we did not carry out any experiment using samples belonging to this group. We added a note below table 1 (line 123) to explain it better.


Reviewer #2: The authors have substantially revised the manuscript to address my previous concerns. The manuscript is significantly improved and I consider it to be worthy of publication.

We thank the reviewer.
